# Qualitative evaluation of a coaching and leadership program for early-stage researchers in KL2 and T32 training programs

**DOI:** 10.1017/cts.2026.10740

**Published:** 2026-04-22

**Authors:** Lauren Jodi Van Scoy, Erika VanDyke, Marie L. Boltz, Dale Fallon, Jessica Petrie, David Rábago

**Affiliations:** 1 Department of Medicine, Penn State College of Medicine, Hershey, PA, USA; 2 Ross and Carol Nese College of Nursing, The Pennsylvania State University, Hershey, PA, USA; 3 The Communication Gym, LLC, Mechanicsburg, PA, USA; 4 The Department of Family and Community Medicine, Penn State College of Medicine, Hershey, PA, USA

**Keywords:** Coaching, leadership development, career development, postdoctoral fellows, early-career faculty

## Abstract

**Background:**

Early-stage clinical and translational researchers require not only technical expertise but also leadership and communication skills for long-term success. Many training programs lack structured approaches to building these essential “soft skills.”

**Objective:**

To evaluate the impact and perceived value of a structured coaching and leadership program for trainees in KL2 and T32 programs.

**Methods:**

This qualitative evaluation assessed a Coaching and Leadership Program (CLP) that include individualized coaching and group workshops incorporating leadership development and the DISC behavioral communication model (Dominance, Influence, Steadiness, and Conscientiousness). Semi-structured interviews were conducted with twelve KL2 and postdoctoral T32 trainees between July and August 2025. Transcripts were analyzed using descriptive content analysis and inductive coding by two analysts in MAXQDA.

**Results:**

Five key themes emerged: 1) Both T and K trainees consistently described the CLP as a broadly positive and beneficial experience; 2) Coaching helped trainees build concrete organizational strategies, particularly around time management and logistical processes; 3) The CLP helped build trainees’ confidence and professional identity, especially around communication with mentors, bosses, or their team; 4) The CLP was perceived as complementary and well integrated within KL2 and T32 training, particularly by addressing “soft skills” missing elsewhere; and 5) Participants recommended stronger orientation, more opportunities for practical skill-building during sessions, and offering greater variety and choice in coaches.

**Conclusions:**

The CLP complements scientific training for early-career translational researchers. Trainees gained practical tools for team management, conflict resolution, and strategic planning, while benefiting from a confidential space for professional growth. Findings suggest coaching is a valuable enhancement to training programs for developing translational research leaders.

## Introduction

Developing the next generation of clinical and translational scientists requires rigorous technical training and acquisition of leadership, communication, and team management skills [1–3]. While early-career researchers often enter competitive training programs with strong content expertise, many have limited experience in areas such as managing teams, navigating conflict, and advocating effectively within complex institutional environments [4,5]. Designing professional development curricula for such trainees is challenging because of the heterogeneity of their backgrounds, research disciplines, and future career paths [5,6].

Mentorship has long been recognized as a central component of research training, offering guidance on content expertise and career development [7,8]. Mentorship is defined by the National Academies of Sciences Engineering, and Medicine [9]: “*Mentorship is a professional, working alliance in which individuals work together over time to support the personal and professional growth, development, and success of the relational partners through the provision of career and psychosocial support*.” However, mentoring alone may not adequately address gaps in leadership or communication skills due to the competing and sometimes incompatible demands placed on mentors and a lack of dedicated mentoring time for many faculty mentors [7,10]. In practice, mentors often juggle multiple demands and receive limited formal preparation for their role [8]. As such, mentoring relationships, while invaluable, may not fully meet the developmental needs of trainees [11]. Over the past two decades, professional coaching has emerged as a complementary approach to mentoring in support of career development [12–14]. Coaching is defined by the International Coaching Federation (ICF) as partnering with clients in a thought-provoking and creative process that inspires them to be their best personal and professional selves [15]. Others have described coaching as a special, sometimes reciprocal, relationship between (at least) two people who work together to set professional goals *and* achieve them [16]. Coaching involves a structured, confidential, and individualized process focused on fostering reflection, enhancing leadership capacity, and building practical skill [16]. Although coaching and mentoring share some features, coaching differs in its emphasis on process over content, self-directed goal-setting, and sustained accountability, while mentoring is described by the ICF as a “seasoned guide” that shares wisdom and develops a long-term relationship [17]. Coaching is increasingly used in business, medicine, and academic contexts [14,18], yet evidence regarding its effectiveness in structured training environments for clinical and translational research trainees remains limited [13,19,20].

Although challenging to assess impact and “return on investment” per se, the benefits of coaching programs have been widely recognized across a range of industries, including healthcare and academia [21,22]. Several recent randomized controlled trials demonstrated reduced clinician burnout among those who received coaching [23,24]. More broadly, coaching has been associated with increased career engagement, and there is growing consensus that such programs may contribute to enhanced productivity, improved workplace satisfaction, and stronger team functioning [21]. These observations highlight the pressing need for a more robust evidence base on the design, implementation, and determination of key outcomes (and their measurement), and evaluation of coaching programs.

Early-career scientists from KL2/K12 and T32 programs are in key developmental stages supported by NIH and other federally funded programs and thus stand to benefit from structured coaching. In our experience, many trainees require and/or request additional support in real-world application of leadership and managerial skills that coaching can provide. However, questions remain about optimal integration of coaching into training structures, the impact coaching has on trainees, and how it complements mentorship and other curricular components. The purpose of this study was to explore the impact and perceptions of a structured Coaching and Leadership Program (CLP), which involved both individualized coaching and workshop-based leadership sessions for trainees in Penn State’s KL2 and T32 programs. We sought to understand how coaching was experienced, what skills and competencies were gained, how coaching integrated with other training elements, and what improvements might enhance its value for future cohorts.

## Methods

### Participants and recruitment

This evaluation was conducted at Penn State College of Medicine among participants in two programs: National Research Service Award (NRSA)/Health Resources and Services Administration (HRSA)-funded T32 fellowship (T32HP42015, 2021–2026), Ruth L. Kirschstein National Research Service Award Institutional Research Training Grant; “Fellowship”); and the National Center for Advancing Translational Sciences (NCATS)-funded KL2 early-career development program supported by the Penn State Clinical and Translational Science Institute (KL2TR002015, 2016–2026; “KL2”). Current trainees who were enrolled in either program from 2022–2025 (*n* = 16) were contacted via email to participate in a 1:1 interview with an external qualitative interviewer. The final sample included twelve participants.

### T32 program description

The T32 Fellowship trains postdoctoral fellows (hereafter called “trainees”) across the biomedical spectrum (MD, DO, PhD, DrPH, etc.) to become independent researchers with a primary care focus. Research may span traditional primary care topics, with an emphasis in the current cycle on substance use and mental health. Methodological rigor, team science, and translational relevance are core components. Housed in the Penn State College of Medicine Department of Family and Community Medicine, the T32 program is also led by faculty from Academic General Pediatrics and General Internal Medicine, with additional mentorship and instruction from experts across the College and University. Trainees are nationally recruited through a merit-based process aligned with HRSA criteria. Five trainees are supported annually in two- to 3-year, full-time research positions (1.0 FTE), with salary determined by federal postdoctoral experience guidelines. In addition to the CLP described below, training includes three core elements: hands-on research, individualized coursework, and a structured seminar series. Research activities begin at selection, with early identification of mentors, research focus, and coursework needs. Fellows develop Individualized Development Plans (IDPs) and mentor–mentee compacts, reviewed every 6 months. Coursework is tailored to address knowledge gaps or lead to certificates or degrees (e.g., public health, clinical research, translational science). Fellows attend weekly 2-hour seminars (∼40/year) covering core topics such as research question development, methodologies, data stewardship, abstracts/manuscript/grant-writing, and career development. The CLP was integrated into the existing T32 curriculum and consisted of five seminars and seven individualized meetings.

### KL2 program description

The KL2 program supports early-stage investigators (hereafter called “trainees”) with 75% protected research time, research funds, tuition support, and structured mentorship over a 2- to 3-year period. Four to eight junior faculty are selected annually through a competitive application process. Eligible applicants include full-time Penn State faculty with a clinical or research doctorate (MD, DO, PhD, MD/PhD, PharmD, DrPH), typically at the assistant professor level, with an emphasis on clinician scientists. The KL2 program delivers a comprehensive translational science curriculum featuring weekly 2-hour sessions, individualized mentor meetings, private coaching, and community-engaged research experiences. Trainees receive training in theoretical and methodological foundations necessary to conduct rigorous, impactful research. Core components include leadership and coaching (described below), grant-writing workshops, and translational science seminars. The grant-writing series addresses all components of NIH proposals – from specific aims and research strategy to practical tips on engaging NIH Program Officers. Successful proposal excerpts from trainees and faculty are critiqued during sessions. The translational science seminar series covers methodology, data science, regulatory affairs, implementation science, ethics, statistical strategies, community-engaged research, and dissemination. Sessions combine didactic instruction with case-based discussion and applied exercises, delivered by experienced faculty from across the institution.

### CLP design

The CLP was implemented by a professional leadership and communication coach, co-author Dale Fallon of The Communication Gym. Mr. Fallon, an executive communication coach with over 25 years of experience coaching leaders across broad industries, served as the coach for both the KL2 and T32 programs. Mr. Fallon’s background includes coaching for the biomedical research community, providing coaching to more than 50 academic trainees at various career stages, including graduate students, junior faculty, and senior investigators. He has logged over 15,000 hours in his 25 years of coaching (master certification requires 2500 hours) and is well versed in the ICF frameworks. Existing content from The Communication Gym incorporating leadership and communication trainings were integrated into the KL2 and T32 programs in partnership with the program directors. The entirety of the CLP was mandatory for both programs, however, program leaders did not force additional coaching sessions if the trainee did not wish to continue them past the first coaching session (as “mandatory” coaching is unlikely to be productive). That said, all trainees but 1 KL2 scholar utilized all their available sessions. Funding was provided by from the T32 and KL2 programs’ grants and/or internal funds. The scale of both the T32 and KL2 programs (including the number of coaching sessions) was based on resource availability of the two programs. The workshop series was customized in collaboration with the programs’ leadership to be responsive to the programmatic needs and career stages of trainees.

The CLP was designed as a structured but individualized complement to the technical and scientific training provided by the KL2 and T32 programs. Each trainee participated in a series of one-on-one sessions with the coach (up to 3 for the KL2s and up to 7 for the T32s; numbers chosen based on budget considerations), in addition to participation in a broader leadership development seminar series. Individual coaching sessions were confidential, trainee-driven, and flexibly scheduled, either virtually or in person. Sessions typically lasted 45–60 minutes and focused on challenges or developmental goals identified by the trainee. Coaching was delivered by a single experienced coach who began with a structured, in-person intake to clarify the client’s objectives, challenges, communication style (including assessment and coaching based on DISC [Dominance, Influence, Steadiness, and Conscientiousness] profiles), and personal and professional roles. Subsequent sessions were guided by the trainee’s agenda but remained firmly oriented toward the operating goals established at intake, with the coach identifying coaching gaps and collaboratively shaping focused strategies and action steps. The coach consistently brought the conversation back to the broader objectives to ensure continuity, accountability, and meaningful progress. Topics included communication with mentors and supervisors, navigating team dynamics, negotiating workload expectations, setting boundaries, and strengthening leadership presence.

The group workshops were customized for the KL2 and T32 programs in partnership with the program directors (Table 1) and had required attendance. Sessions were interactive and applied, combining brief didactic content with small group, guided reflection, and team-based problem-solving. While the leadership sessions provided shared frameworks and opportunities for group reflection, the individual coaching sessions created a personalized space to apply those concepts in real time to each trainee’s context.

**Table 1.** Description of the coaching programs

Session format	KL2 program	T32 program
Individualized coaching	• 1:1 sessions • 45–60 min each • Scheduled flexibly by trainees • 3 sessions per trainee	• Seven 1:1 coaching sessions • 45–60 min each • Scheduled flexibly by trainees • 7 sessions per trainee
Group workshops	Six 2-hour workshops: **Session 1**: Clarifying your role as a leader **Session 2**: Building your team **Session 3**: Motivating teams using the DISC communication styles **Session 4**: Managing and growing your team’s capacity **Session 5**: Preparing for successful collaborations using team science **Session 6**: Communication skills of resilient leaders	Five 2-hour workshops: **Session 1:** Understanding communication and leadership styles **Session 2**: Making time for conversations that count **Session 3:** Preparing for successful collaborations **Session 4:** Coaching your research team **Session 5:** Optimizing research output

Notably, the CLP had a focus on communication training, using the DISC behavioral model (Dominance, Influence, Steadiness, and Conscientiousness) which was integrated into both group workshops and individual coaching sessions. The DISC is a widely used framework [25] that categorizes behavioral tendencies into four distinct communication styles (D, I, S, and C). A goal is for trainees to learn to recognize these styles, thereby gaining insight into how colleagues may differ in their approaches to communication, leadership, conflict management, and team collaboration. The training emphasized self-awareness, understanding one’s own style and default tendencies, and tailoring communication approaches to better align with others. Each trainee completed a personalized DISC assessment and then received tailored feedback during coaching sessions. In group workshops, trainees practiced recognizing DISC patterns in others and explored strategies for applying these insights to real-world scenarios, such as leading research teams, negotiating with mentors, and managing conflict.

Although multiple assessments exist, we selected the DISC because it offers a practical framework for navigating interpersonal dynamics across different environments and audiences – and the CLP coach has extensive experience incorporating this particular assessment into programming. Further, the DISC is supported by decades of organizational use, refinement, and technical validation [25].

### Qualitative interviews

Semi-structured interviews were conducted with a total of 12 trainees of the KL2 and T32 programs between July and August 2025. An interview guide was developed around five domains: (1) CLP experience and overall assessment; (2) coach relationship and effectiveness; (3) perceived impact of both coaching and leadership sessions; (4) integration with existing KL2 and T32 offerings; and (5) suggestions for CLP improvement. Example interview questions included, “What aspects of coaching did you like best?,” “What specific skills or competencies did you develop through the CLP?,” and “Were there any conflicts or redundancies between the CLP and other mentoring you received? If so, can you please explain?” Interviews were conducted via Zoom, audio-recorded with participant consent, and professionally transcribed.

### Data analysis

A descriptive content analysis was conducted on transcribed interviews (*n* = 12). Research team members with qualitative research expertise (EV and LJV) reviewed all transcripts to inductively create categories, codes, and sub-codes that described data to develop the final codebook. Analysts used the final codebook to code the transcripts and discuss coding styles to ensure coding consistency. The analysts first coded 20% of the dataset and met to discuss codes and adjudicate as necessary. MAXQDA (v. 24; VERBI GmbH, Berlin, Germany) was used to organize and code the data [26]. To maintain coding rigor, inter-rater reliability between analysts was calculated (*K* = .79). After calibration, the analysts split the remaining transcripts and coded the rest of the data set. The study team then reviewed coding to develop themes. Themes and quotes are described in “Results.”

### Ethical considerations

This evaluation was determined to be not human subjects research by Penn State University Institutional Review Board. All participants provided consent for recording and inclusion of de-identified quotes. Given the small sample size, to maintain anonymity, interviews from both KL2 and T32 participants were pooled to prevent inadvertent participant identification.

## Results

### Participant characteristics

Participation was offered to all trainees in the KL2 (*n* = 10) and T32 (*n* = 6) programs, with the majority identifying as female (81%) and White (81%; Table 2). Educational backgrounds varied, with representation across MD, PhD, DrPH, and dual-degree training pathways. To protect individual confidentiality, characteristics of non-participants were not collected. Twelve trainees participated in qualitative interviews (7 KL2 and 5 T32), representing a subset of the total potential cohort (*N* = 16).

**Table 2.** Program participant characteristics (full cohort *N* =16)

Characteristics	KL2 trainees *n* = 10 in cohort 7 participated in interviews	T32 trainees *n* = 6 in cohort 5 participated in interviews
**Gender**		
Male	2 (20.0%)	1 (16.7%)
Female	8 (80.0%)	5 (83.3%)
**Age**		
Mean (SD)	40.5 (5.4)	41.5 (6.3)
**Race**		
Asian	2 (20/0%)	0
Black/African American	0	0
White	7 (70.0%)	6 (100%)
Other/multiple races	1 (10.0%)	0
**Ethnicity**		
Hispanic/Latino	1 (10.0%)	2 (33.3%)
Not Hispanic/Latino	9 (90.0%)	4 (66.7%)
**Education**		
MD	4 (40.0%)	1 (16.75)
PhD	6 (60.0%)	2 (33.3%)
DrPH	0	2 (33.3%)
Dual degree MD	0	1 (16.7%)

### Content analysis

Five themes emerged from the analysis (Table 3):

**Table 3.** Summary of themes from trainee interviews

**Theme 1**	Both T and K trainees consistently described coaching as a broadly positive and beneficial experience that they would recommend to others.
**Theme 2**	Coaching helped trainees build concrete organizational strategies, particularly around time management and logistical processes.
**Theme 3**	The curriculum helped build trainees’ confidence and professional identity, especially around communication with mentors, bosses, or their team.
**Theme 4**	Coaching was perceived as complementary and well integrated within KL2 and T32 training, particularly by addressing “soft skills” missing elsewhere.
**Theme 5**	Participants recommended better framing of the curriculum, more opportunities for practical skill-building during sessions, and offering greater diversity and choice in coaches.

**Theme 1: Both T and K trainees consistently described coaching as a broadly positive and beneficial experience that they would recommend to others.** Overall, trainees in both groups had favorable impressions about the CLP and found value in the sessions describing them as “*helpful*,” “*useful”*, and “*worthwhile”*, even at this early stage of their academic careers.

*I think it’s a good thing to have this now in your early career, because this is really helpful just getting the bases ready for your future to build on it… It’s been very helpful, eye opening, and I think it’s good if you start now implementing these things.* (Interview 9, T32 Trainee)

When asked about their impressions of the CLP, trainees commonly emphasized the benefits of being explicit in defining their goals and vision, particularly in the context of the coaching sessions. One participant appreciated the focus on defining their “end goal,”

*I think the one-on-one sessions has been helpful. Just thinking about what your end goal is going to be and how to navigate through everything to get there. It’s been helpful in the sense that even I sometimes do not know where my end goal wants to be. And talking with Dale [the coach] has been helpful in manifesting on different steps to get where I want to be focusing on short term goals and how that fits within your long term goals and also advocating for what you want to do and how that would fit into what you see you want to do in the future.* (Interview 9, T32 Trainee)

Similarly, K trainees found goal and mission-setting to be a positive aspect of the sessions, particularly when related to their growing research program and maintaining focus on goal attainment. For example, a K trainee said,

*The first few meetings we had in the year were focused on my vision, as a principal investigator and the mission statement for our lab, which was not something I had thought about… and then discussed ways of what do I want to -- how do I measure whether or not I’m succeeding at achieving those goals and Dale [the coach] gave me some practical strategies that then I practiced implementing.* (Interview 8, KL2 Trainee)

Several trainees found the flexible nature of the sessions and agenda focused on what was happening in their lives and career in the moment as a helpful approach to help connecting them back to their overall vision.

*I think the other piece that has been really helpful for me is that I am very much like a long-term vision person, and Dale [the coach] was really good at saying things like how do you connect this thing that’s right in front of you back to that long term vision? And always making sure that you’re kind of toggling back and forth between where you are and where you’re going… a lot of our conversations really centered around that and that was really helpful for me.* (Interview 12, KL2 trainee)

Multiple trainees noted that while they were skeptical of coaching at first, the experience exceeded expectations. A T32 trainee said,

*I used to think that it was not a very serious kind of thing. I feel like lots of people improvise themselves as coaches…I was a bit skeptical. But at the same time, I did recognize that the program is led by smart people who go by evidence and all that…I think I’ve been happily surprised of, I’m not sure how to describe the impact, but it’s been enjoyable, and I think I’ve learned stuff about myself…It’s given me someone that can hold a mirror, but that also has no [influence on] my career or my reference letters…* (Interview 11, T32 Trainee)

**Theme 2: Coaching helped trainees build concrete organizational strategies, particularly around time management and logistical processes.** While trainees described coaching as supporting their professional development in multiple ways, they particularly appreciated the curriculum’s focus on tangible strategies for managing research programs including attention to team infrastructure and improving time management. These topics were commonly described as highly useful and appreciated by both groups of trainees. They consistently mentioned that the opportunity to hone these skills in an applied setting, one-on-one with Mr. Fallon, was particularly valuable. For example, a KL2 trainee described how coaching helped increase their sense of clarity in their roles and fostered greater awareness of the importance of efficiency and prioritization when leading a research lab.

*I think a lot of the work, anything related to time management or organization, that’s to help make me more efficient and thus productive and successful at that mission statement. … the intent was to really help communicate to the people who work for me why we’re doing this work and continue to have them be motivated and figure out myself what do I prioritize when running a lab to help achieve the things I’m most interested in achieving? I think it’s been helpful in that respect of time management efficiency and then being a more effective leader to lead a lab.* (Interview 8, KL2 trainee)

Another participant emphasized how useful it was to have Mr. Fallon as a sounding board for discussing organizational structures unique to their research setting:

*We talked a lot more about organizational structures, because I run an independent lab at [campus]… It was a lot about managing that time, and efficiency, like automating processes, and figuring out that kind of stuff. I think that was probably the most useful piece, just the independent sounding board to flesh ideas out about the organizational structure, and interpersonal dynamics…* (Interview 2, KL2 Trainee)

A T32 trainee emphasized that they learned how logistical resources and project management tools (such as Gantt charts) can help in project management, and also had real implications for progress and productivity. One T32 trainee shared a key point:

*…it’s not about your ability to do things. It’s really about the systems that you have in place to do things that actually going to get you places, and I think that’s something very valuable as well. And I think that’s all where the Gantt charts come in… it’s like a system for tracking projects and tracking things to do, and I think that is really helpful.* (Interview 4, T32 Trainee)

**Theme 3: The CLP’s focus on communication styles helped build trainees’ confidence and professional identity, especially around communication with mentors and bosses.** Participants also identified as a strength the focus on interpersonal communication by providing a dedicated space to consider communication skill-building, especially related to navigating conversations with mentors and bosses. Several participants recounted using coaching sessions to prepare for difficult conversations:

*I worked through several challenging conversations with Dale [* the coach*] on one-on-one sessions, and I found that to be really helpful to prepare. It’s very interactive, which I appreciate.* (Interview 10, KL2 Trainee)

Several participants noted how the communication focus helped build self-confidence, and the ability to respond more constructively and openly to critical feedback.

*[Coaching has] been really good with self-confidence and learning to respond to criticism, learning how to be a working academic necessarily requires you learn how to do with criticism in general. And the feedback can sometimes be real tough. And so I think that’s also being a good skill that he has helped me develop… be okay with the discomfort*. (Interview 4, T32 trainee)

Others noted that learning about communication styles, particularly through DISC profiles, gave them a framework to better understand and navigate interpersonal dynamics in academic settings. One trainee explained:

*I think one thing that was quite positive was learning the DISC profiles and learning my different communication and leadership qualities, things that I didn’t even know existed. And then, Dale [*the coach*] was able to tailor various scenarios based off my style and was able to give me advice on how to communicate and negotiate with individuals who I was then able to recognize had different DISC profiles. I think that was positive, or maybe the most positive. I’m now thinking about people’s various communication styles in my career when I interact with them…What did Dale [*the coach*] tell me is the best way to negotiate with them? I would say it’s altering my approach to my career in that regard.* (Interview 1, KL2 trainee)

A KL2 trainee noted an encounter with their boss and how one-on-one coaching helped navigate difficult conversations with a dominant personality type.

*I have a tendency to get, bowled over by [my boss] a little bit. And so, it’s been really helpful in thinking through, when I need things from my boss how to approach [my boss] in a way that’s going to be productive [to help me get] things that I need to continue to be successful.* (Interview 12, KL2 trainee)

In sum, the coaching emphasis on communication style and feedback strategies helped trainees feel more equipped to navigate high-stakes interactions that emerged during their training.

**Theme 4: Coaching was perceived as complementary and well integrated with KL2 and T32 training, particularly by addressing “soft skills” missing elsewhere.** Trainees described the coaching intervention as well aligned with the broader goals of their KL2 and T32 programs. Rather than overlapping with existing coursework or mentoring structures, trainees noted how coaching filled a gap by providing targeted support for leadership, communication, and other “soft skills” often absent in traditional research training.

They described the coaching as directly relevant to their day-to-day academic work but that the topics and content went beyond those traditionally taught in past experiences or in the KL2 or T32 programs prior to initiation of the CLP. As one participant explained:

*I think it complemented [the program] really well. Because the program is a training [program]… I think that part of this training is learning not just how to do research, but how to be a researcher*. (Interview 4, T32 trainee)

Another participant similarly shared the importance of these underappreciated skills for long-term success in addition to traditional research skills:

*…the hard skills of doing research are being addressed [in other areas of the curriculums]… This is the soft skills…how do you build your team? How do you get a productive team? How do you stay productive? How do you manage your time? … we neglect the soft skills but at the end of the day… that focus on process, that focus on team, that focus on making sure that everyone is moving forward together and trusting other people to do their jobs … I think all of that has been hugely helpful.* (Interview 12, KL2 trainee)

Overall, trainees’ comments reflected a consistent perception that coaching was not just an “add-on,” but an essential supplement that helped trainees integrate technical expertise with professional self-awareness, team leadership, and strategic communication.

**Theme 5: Participants recommended an orientation framing the role of coaching to better contextualize the program, as well as more opportunities for practical skill-building, and offering greater diversity and choice in coaches.** While participants overwhelmingly valued the coaching experience, several noted opportunities for program improvement, particularly in how the intervention was introduced. One common recommendation was the need for a clearer orientation or framing at the outset. A KL2 trainee noted, “*I think at the beginning, I was a little confused as to what Dale [*the coach*] could offer*.” (Interview 5). Others echoed initial uncertainty and skepticism, suggesting that more explicit guidance could help trainees understand the coaching model and set expectations earlier in the program.

Trainees also expressed a desire for more structured opportunities to practice skills discussed during sessions. While the conceptual and reflective elements of coaching were appreciated, several participants wished for more hands-on application. As one T32 trainee explained:

*I would benefit from them more if they had a greater stronger practice component. Because he’s talking about communication. He’s talking about…organizational skills. He’s talking about setting goals and developing processes and systems … I think they could be improved by adding more practical applications of the theoretical stuff.* (Interview 4, T32 trainee)

Another suggestion was to offer a choice of more than one coach to allow trainees to select a coach who aligned better with their personal and professional needs. Although the individualized nature of coaching was generally well received, some participants raised concerns about coach fit, comfort discussing sensitive issues due to mismatched genders, and the potential value of having a choice. As one KL2 trainee reflected:

*If there was more than one coach, it would be nice. Because if you don’t mesh well with that person, then that’s just tough… and I think having a female option would be good… there are just some things you talk about with a female that you wouldn’t necessarily feel as comfortable talking to a male about.* (Interview 3, KL2 trainee)

Collectively, suggestions for improvement centered on enhancing access and tailoring the coaching experience – such as offering more practical skill-building and greater coach diversity – rather than reflecting dissatisfaction with the CLP itself. Notably, no participants from either group expressed overtly negative views or described the coaching as ineffective.

## Discussion

This qualitative evaluation suggests that individualized coaching addressing leadership, communication, and organizational challenges faced by KL2 and T32 research trainees can meaningfully complement traditional mentorship and technical training. Trainees consistently described coaching as a unique and practical opportunity to step outside the technical and methodological focus of their KL2 or T32 programs and to engage in structured reflection about leadership, organizational processes, communication, and career strategy. The sessions provided a confidential space that enabled candid exploration of challenges, while also offering practical tools that could be directly applied to research team management and logistical processes, conflict resolution, and negotiations with mentors and bosses. In this way, coaching served as an important counterbalance to the traditional “hard skills” training offered through KL2 and T32 programs.

Interestingly, several trainees perceived coaching as especially useful during high-stress periods, such as grant deadlines or leadership transitions, suggesting that aligning coaching opportunities with program milestones could enhance impact. The flexibility and responsiveness of the sessions were viewed as critical to their success, underscoring the importance of preserving adaptability even within more structured programmatic frameworks.

Our findings align with prior reports reporting the value of coaching for faculty growth and goal-setting [13]. This evaluation extends that literature by examining a model that used an external coach rather than an internal program member trained in coaching. External coaches may offer greater objectivity, being less influenced by institutional politics or hierarchies, but they may also lack familiarity with institutional culture, resources, and processes, which can limit contextual relevance. In a review of coaching models, Carey et al. concluded that evidence does not support external or internal coaches as inherently superior, as both approaches offer distinct advantages and disadvantages [20]. Consistent with our findings that trainees valued the opportunity to select a coach based on fit and other characteristics, it may be most reasonable for programs to consider a hybrid model that incorporates both internal and external coaches [27].

Another unique feature of the CLP was the integration of the DISC model [28] to help trainees recognize communication styles and adapt their approach across different contexts. This framework was well received, complementing traditional leadership and coaching curricula, and was valued by both T32 and KL2 trainees. DISC role-plays for guiding leadership and communication strategies have been published and may be a useful way to expand use of the framework in a way that allows for more applied learning and practice [29]. The positive perception of our trainees aligns with prior research demonstrating the effectiveness of using DISC for accurate communication self-assessment and team building [30]. Future research should explore how best to incorporate DISC into academic training, including whether its impact varies by career stage and how it might be combined with other leadership development strategies.

Overall, T32 and KL2 trainees reported similarly positive reactions to the CLP. Although some tailoring occurred by career stage in collaboration with KL2 and T32 program directors, the core content and messaging were highly overlapping across the two programs. The shared enthusiasm from trainees suggests the CLP is transferable across developmental stages and could serve as the foundation for a longitudinal coaching model supporting translational scientists as they move through career stages.

From the perspective of the coach, the coaching experience differed somewhat between KL2 and T32 participants. For KL2 trainees, who are early-stage faculty balancing significant research and institutional responsibilities, coaching often emphasized lab and team management, leadership identity formation, and strategic decision-making. In contrast, T32 trainees are postdoctoral trainees, more frequently engaged around communication with mentors, developing confidence, self-advocacy, and managing the demands of training. As the coach observed, KL2 trainees often entered coaching sessions with clearer long-term trajectories and questions about sustaining leadership in high-stakes environments, whereas T32 trainees focused more on immediate interpersonal effectiveness and building foundational professional skills. These observed differences in session content suggest that tailoring coaching more explicitly to developmental stage could enhance the long-term value of the intervention.

Finally, this work has implications for how coaching might be more effectively integrated into structured research training, by providing an early, CLP orientation and framework to forecast themes and tasks of the coaching program, and accessibility to coaches with varied backgrounds and characteristics (although data on gender-matching between coaches and coachees has not been shown to be consistently related to outcomes) [31]. Additionally, while coaching has demonstrated clear benefits for professional development and performance enhancement, it is not without financial cost. Coaching is inherently resource-intensive, often requiring trained and experienced professionals with sustained engagement. As of 2025, highly experienced/certified coaches range from $200 to $500 per hour [32]. Less experienced/uncertified coaches typically range $50 to $150 per hour [32]. To ensure scalability and sustainability, program directors should explicitly plan for coaching expenses during program development and budget forecasting. Institutions, in turn, must acknowledge that while the benefits of coaching and its outcomes are notoriously difficult to quantify [33], it is widely agreed there is likely downstream impact on productivity, retention, and leadership development to justify the investment [34]. Importantly, the coaching field includes a growing number of certified and experienced professionals, making scalability a logistical rather than conceptual challenge. Furthermore, emerging technologies (including development and implementation of artificial intelligence-based coaching tool) hold promise as scalable, cost-effective supplements. While these tools are not substitutes for human coaching, they may augment traditional models by providing accessible, just-in-time support and reinforcement, thereby enhancing overall program reach and efficiency.

Another important consideration is consideration of the optimal “dose” of coaching necessary to achieve meaningful and durable impact. We selected three coaching sessions for K trainees as a pragmatic balance between feasibility, cost, and anticipated benefit. The greater number of sessions provided to T trainees reflected budgetary and structural considerations rather than a fundamentally different coaching model. Prior work experience from our coach suggested that fewer than three sessions is often (but not always) insufficient to move beyond surface-level insight into sustained behavioral change, whereas three sessions allow for initial goal-setting, iterative reflection, and application over time. Importantly, coaching dose was not intended to be prescriptive or uniform; rather, coaching was conceptualized as a dynamic process responsive to individual goals and progress. Three sessions were viewed as a minimally sufficient dose likely to have lasting impact, while also allowing us to assess whether participants would seek or benefit from additional coaching.

Our evaluation has several limitations. The small number of participants from a single institution limits generalizability, however T32 and KL2 programs nationwide have similar funding and structural features across institutions. Our small sample size (and limited number of individual coaching sessions) also prohibits a comprehensive assessments of CLP impact. As such, collaborative studies across institutions are warranted. Further, this study reports data from a “single-coach” program; thus it is difficult to ascertain whether benefits accrue from the CLP itself or from the skill and style of the coach. Since only 12 of 16 trainees participated in the interviews, there is also a potential for selection bias. Additionally, our scholar cohorts were predominantly female, our interviewees were similarly majority female, and the mean age was similar between the two programs (due to an outlier in the T32 program). Thus, the perspectives of non-participants may differ from those who participated, which could limit the interpretation of our findings. Moreover, while trainees provided qualitative examples of how coaching influenced their leadership practices and career decisions, the evaluation did not assess objective outcomes such as grant productivity, publication output, or promotion. Literature reviews have noted the challenges inherent in measuring effectiveness of coaching interventions with “hard outcomes” [18]. Future work could build on these findings by comparing outcomes in T32 and/or KL2 programs randomized to receive, or not receive coaching, similar to prior randomized studies of mentorship [35].

Overall, this evaluation extends evidence that coaching offers meaningful benefits for KL2 and T32 trainees, supporting the development of skills that are not otherwise addressed in traditional research training. Coaching’s focus on reflection, DISC-style communication, and leadership provides a critical complement to the technical skills emphasized in translational science programs. By refining integration strategies and addressing areas for improvement, coaching has the potential to become an enduring and impactful feature of career development for early-stage investigators.
